# Management of Colorectal Cancer Liver Metastases

**DOI:** 10.3390/cancers16020420

**Published:** 2024-01-19

**Authors:** Jakob A. Durden, Ioannis A. Ziogas, Dimitrios P. Moris, Ana L. Gleisner

**Affiliations:** 1 Department of Surgery, University of Colorado Anschutz Medical Campus, Aurora, CO 80045, USA; jakob.durden@cuanschutz.edu (J.A.D.); ioannis.ziogas@cuanschutz.edu (I.A.Z.); ana.gleisner@cuanschutz.edu (A.L.G.); 2 Department of Surgery, Duke University Medical Center, Durham, NC 27710, USA

Ivey et al. have accomplished a nuanced, encompassing review of the contemporary management of colorectal liver metastasis (CRLM) from a surgical perspective. Their work emphasizes the importance of timely surgical evaluation to maximize the benefits of current resection strategies—which frequently need to be individualized based on the patient’s health status, location of the primary tumor, tumor burden and tumor biology [1]. Although tumor biology cannot be precisely estimated, the presence of BRAF mutations and RAS mutations, particularly those associated with TP53 and SMAD4 mutations, is associated with worse outcomes following liver resection [2,3]. In addition, the impact of immunotherapy on the management of patients with CRLM and microsatellite instability is yet to be determined. And while no mutational status is an absolute contraindication to surgical resection, these mutations should be considered, along with other critical factors, when the surgical treatment of CRLM is being discussed.

In particular, this review suggests that all cases involving CRLM should be referred to a multidisciplinary clinic (MDC) for review by a team including a liver surgeon specializing in hepatic tumor resection for a case-by-case review of the feasibility of resection. Indeed, a contemporary meta-analysis has found improved overall survival in stage IV colorectal cancer patients evaluated in MDCs [4]. Unfortunately, such services tend to be limited to resource-rich, high-volume, experienced liver surgery centers. A study on Medicare beneficiaries showed that only a minority of patients with CRLM ultimately received liver-directed therapy (LDT) [5]. Additionally, patients who were black, female, socially vulnerable or lived further from specialized centers were less likely to undergo LDT [5], suggesting access to care may be one of the barriers. Thus, in addition to educating stakeholders on the benefits of MDC evaluation for patients with CRLM, there needs to be an effort to improve access to MDCs, such as by expanding telemedicine services and financial support for disadvantaged patients.

The authors include data on liver transplantation (LT), a treatment option that has increasingly been proposed for patients with unresectable CRLM. As they point out, long-term results from the SECA-II study are encouraging, with an estimated overall survival >80% in 5 years [6]. However, there are ethical concerns regarding the use of livers from deceased donors for this indication, especially in North America, where grafts are still scarce. Living donor liver transplantation (LDLT) can be used to expand the donor pool and has been proven feasible and safe for these patients; preliminary data from the University of Toronto on LDLT in qualifying patients with unresectable, bilobar CRLM showed 3-year survival rates of 100% [7]. Obviously, the use of LDLT presents its own ethical dilemma, as the risk to the donor, as low as it may be, it is not justifiable if there is no survival benefit for the patient. In addition, these patients were highly selected, which suggests some of the benefits seen with LT may be due to the selection of patients with a better tumor biology. Several randomized controlled trials comparing LT to systemic chemotherapy in patients with unresectable CRLM are currently ongoing. At the same time, there has been renewed enthusiasm for the use of intra-arterial chemotherapy through a hepatic artery infusion pump in patients with unresectable CRLM thanks to high rates of conversion to resectable disease in patients treated with intra-arterial chemotherapy and modern systemic chemotherapy [8]. A randomized controlled trial comparing intra-arterial chemotherapy and systemic chemotherapy to systemic therapy alone has just been activated. Within the next decade, we should have more robust data on the benefits of these two modalities in the treatment of unresectable CRLM.

Finally, the authors describe some important technical concepts that should be well known by surgeons treating patients with CRLM: parenchyma-sparing hepatectomy and the minimally invasive approaches. Parenchyma-sparing hepatectomy is typically preferred over anatomic resection due to the decreased risk of postoperative liver failure and increased rates of repeat liver resection in the event of a liver recurrence. In a recent meta-analysis including 22 studies and 7228 patients, compared to anatomic resections, parenchyma-sparing techniques were associated with decreased operative time, blood loss, and 90-day mortality (risk ratio [RR] = 3.08, *p* < 0.001). Anatomic resections were associated with lower positive resection margin rates (RR = 0.77, *p* = 0.024) and intrahepatic recurrence (RR = 0.90, *p* = 0.021) [9]. However, long-term oncologic outcomes, including overall survival, were equivalent between the two groups. Notably, these data are based only on observational studies; no randomized controlled trials comparing the techniques have ever been performed. In the absence of high-quality evidence, parenchyma-sparing hepatectomy should be chosen over anatomic resection when there is a reasonable chance all disease will be resected with negative margins, as well as in patients at higher risk of postoperative liver failure. On the other hand, the minimally invasive approach is increasingly being used for liver resection and, as in other surgeries, has been associated with reduced blood loss and shorter length of stay [10,11,12,13,14]. A recent systematic review and meta-analysis including three randomized controlled trials comparing the minimally invasive approach with open surgery for the resection of CRLM showed lower complication rates in patients who underwent the minimally invasive approach, with comparable disease-free and overall survival rates [15]. Given the current evidence, minimally invasive liver resection should be preferred if anatomically feasible. Nevertheless, the resection of multiple liver lesions through the minimally invasive approach is technically challenging. Thus, the increased utilization of the parenchyma-sparing approach may hinder the implementation of the minimally invasive approach in patients with CRLM. Safety and long-term oncologic outcomes should dictate the type of resection that is appropriate for each patient. Only then should the surgeon decide on the feasibility of proceeding with the minimally invasive approach.

In summary, Ivey et al. have provided an excellent summary of the current options for surgical treatment of CRLM. Hepatic resection is the standard of care in patients with resectable disease and should be performed through a parenchyma-sparing approach when oncologically appropriate and minimally invasively if feasible. For those with unresectable CRLM, treatment recommendations should be determined by a multidisciplinary team, including an experienced liver surgeon, to confirm whether the disease is truly unresectable. Intra-arterial chemotherapy may increase the chances of conversion to resectable disease and can be considered in these patients. For highly selected patients with unresectable disease, LT may be an option, especially if there is no shortage of donated livers. Barriers to liver resection and to MDC evaluation should be further explored and addressed.
